# The PucR-type activator IppR regulates 2-isopropylphenol hydroxylation in strain *Rhodococcus* sp. D-6

**DOI:** 10.1128/aem.01277-26

**Published:** 2026-08-06

**Authors:** Qian Zhu, Kangning Wei, Yike Lyu, Qi Xie, Kaihua Pan, Qian Li, Weihao Zhu, Zhijian Ke, Jiguo Qiu, Mingliang Zhang, Junqiang Hu, Shang Dai, Qing Hong

**Affiliations:** 1 Department of Microbiology, College of Life Sciences, Nanjing Agricultural University, Key Laboratory of Agricultural and Environmental Microbiology, Ministry of Agriculture and Rural Affairs 70578, Nanjing, People's Republic of China; 2 College of Biological and Chemical Engineering, Jiaxing University 66559, Jiaxing, People's Republic of China; 3 Research Center for Planetary Health, Jiaxing University 66559, Jiaxing, People's Republic of China; University of Milano-Bicocca, Milan, Italy

**Keywords:** *Rhodococcus *sp. D-6, isoprocarb degradation, two-component 2-isopropylphenol monooxygenase gene *ippA1A2*, transcriptional regulation, PucR-type regulator

## Abstract

Although genes involved in isoprocarb (IPC) degradation, including the IPC hydrolase gene *ipcH* and the 2-isopropylphenol (IPP) hydroxylation gene cluster *ippA1A2*, have been identified in *Rhodococcus* sp. D-6, the transcriptional regulatory mechanism remains unclear. In this study, we characterized a PucR-type transcriptional regulator, IppR, which controls the inducible expression of the *ippA1A2* cluster. Gene disruption and complementation analyses revealed that *ippR* was essential for activation of *ippA1A2* expression in response to IPP in strain D-6. Quantitative reverse transcription-PCR (qRT-PCR) and promoter activity assays demonstrated that IPP was the effector molecule of IppR. Electrophoretic mobility shift assay (EMSA) showed that IppR bound to a 24-bp motif in the promoter region, with the CC/CG box within this motif identified as the critical site for IppR binding. Moreover, IppR residues likely involved in IPP binding include Val188, Leu192, Val218, Trp257, Trp259, His297, and Ala300, along with Lys382 and Thr384, which are predicted to be essential for DNA recognition. This study elucidates the regulatory mechanism of IPC degradation in strain D-6, and the identification of IppR expands the diversity of regulatory models within the PucR-type regulator.

**IMPORTANCE:**

Carbamate insecticides pose a potential threat to the environment and human health. Bacteria play an important role in the biodegradation of these compounds. *Rhodococcus* sp. D-6, which is capable of degrading isoprocarb (IPC), a representative carbamate insecticide, was isolated in our previous study. The expression of the IPC hydrolase gene *ipcH* was constitutive, while the expression of the *ippA1A2* cluster responsible for hydroxylation of 2-isopropylphenol (IPP), the hydrolysis product of IPC, was inducible. The present study functionally characterized the PucR-type transcriptional regulator IppR, which activates the transcription of the *ippA1A2* cluster to mediate IPP hydroxylation in strain D-6. This study advances our understanding of the regulatory mechanisms underlying IPC degradation.

## INTRODUCTION

Isoprocarb [2-(1-methylethyl)-phenol methylcarbamate, IPC] is a representative carbamate insecticide widely used for pest control in agriculture and forestry (1, 2). Due to its extensive application and chemical stability, IPC residues have been detected in various environments (3–5). IPC exerts its insecticidal activity by inhibiting the activity of acetylcholinesterase, a key enzyme involved in neural signal transmission (6). Therefore, residual IPC in the environment poses potential hazards to humans and non-target organisms (7–10). IPC has been classified as a moderately toxic compound by the World Health Organization (WHO). Investigating the microbial degradation mechanism of IPC not only is of theoretical significance but also holds potential for bioremediation of IPC-contaminated environments.

To date, several IPC-degrading strains have been reported, including members of the genera *Rhodococcus* (11), *Lactobacillus* (12), *Streptococcus* (12), *Acinetobacter* (13), *Sphingobium* (14), and *Bacillus* (15). Among them, the IPC degradation mechanism of *Rhodococcus* sp. D-6, a strain isolated in our laboratory, has been extensively characterized (11). In strain D-6, IPC is first hydrolyzed by the hydrolase IpcH to produce 2-isopropylphenol (IPP). IPP is subsequently hydroxylated by the two-component flavin-dependent monooxygenase IppA1A2 to generate 2-isopropylhydroquinone (11, 16). The resulting 2-isopropylhydroquinone undergoes sequential reactions, including oxidative ring opening, dehydrogenation, and reduction, eventually entering the tricarboxylic acid cycle as a carbon and energy source for the growth of strain D-6 (16). Notably, strain D-6 can utilize IPP, the hydrolysis product of IPC, as its sole growth substrate (16). However, the regulatory mechanism of IPC degradation in strain D-6 remains unclear.

PucR family transcriptional regulators participate in diverse physiological pathways, including amino acid metabolism, fatty acid biosynthesis, purine metabolism, and antibiotic biosynthesis (17–26). They play essential roles in bacterial central metabolism and environmental adaptation. Unlike PucR from *Bacillus subtilis* 168, which functions as both an activator and repressor of the purine catabolic pathway, all other characterized PucR-type regulators act exclusively as transcriptional activators (17–26). PucR family members share two conserved structural domains: a GGDEF-like domain and a PucR-type C-terminal helix-turn-helix (HTH) domain. The GGDEF-like domain is widely present among functionally characterized PucR-type regulators, except for GabR, a regulator of 4-aminobutyrate utilization in *Corynebacterium glutamicum* ATCC 13032 (22, 23). This domain has been hypothesized to mediate effector binding by Megalizzi et al., but experimental validation is currently lacking (23). The C-HTH domain of the PucR family, which binds promoter DNA to modulate transcription of target genes, exists in all functionally characterized PucR family regulators (19, 22, 23).

Previous studies showed that the *ipcH* gene is constitutively expressed in strain D-6, while the *ippA1A2* cluster is inducible (11, 16). In this study, the PucR-type regulator IppR was identified as the transcriptional activator of the *ippA1A2* cluster in response to IPP in strain D-6. The regulatory mechanism was investigated, including the transcriptional units, DNA-binding sites, effector molecule, and key amino acids essential for effector binding and DNA recognition.

## RESULTS

### IppR positively regulates IPP degradation in strain D-6

**Fig 1:**
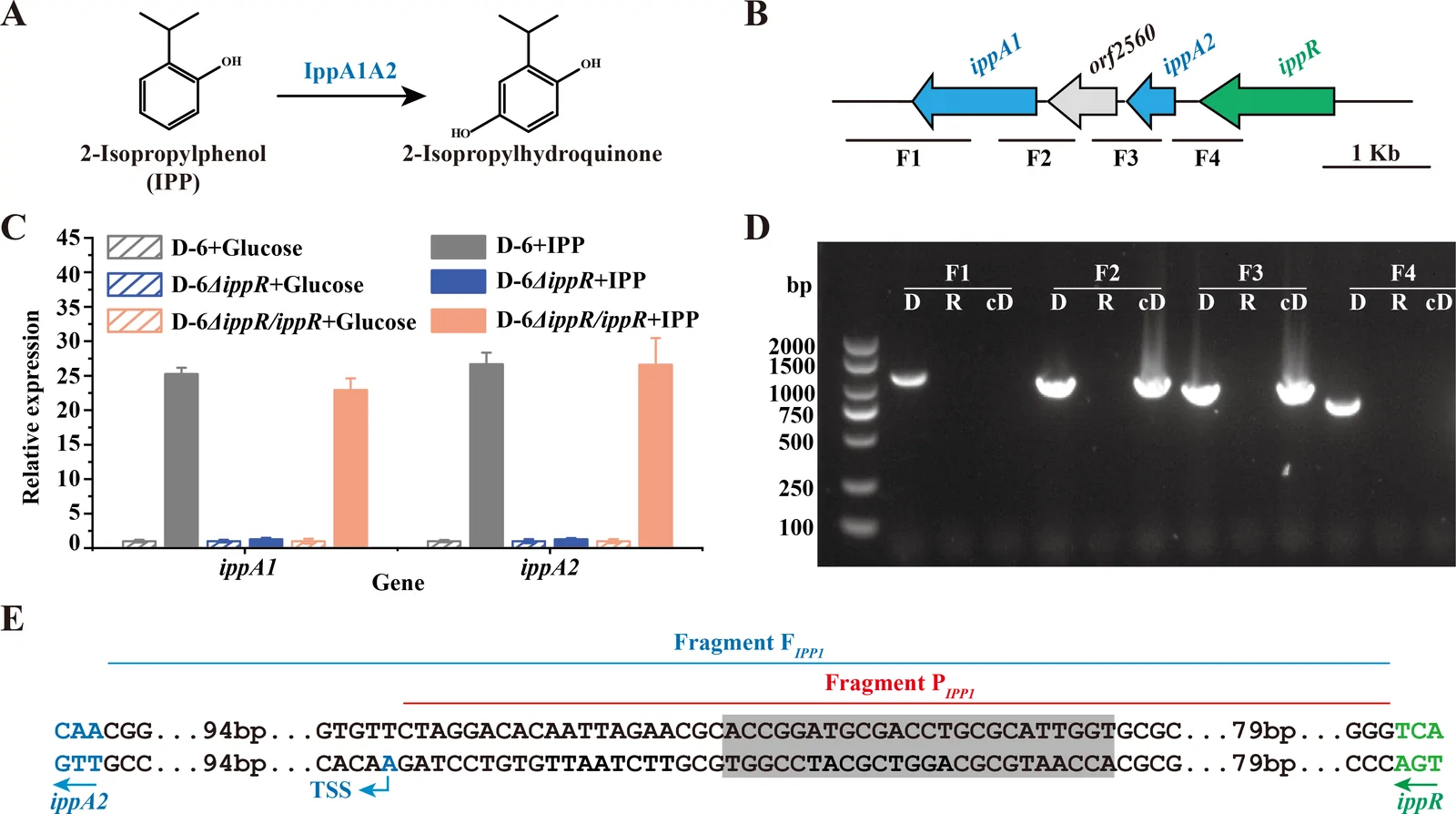
(**A**) Hydroxylation of 2-isopropylphenol (IPP) to 2-isopropylhydroquinone by the two-component monooxygenase IppA1A2. (**B**) Schematic diagram of the *ippA1A2* gene cluster in strain D-6. The amplification fragments for transcriptional unit evaluation are shown under the gene cluster as lines. (**C**) Transcriptional analysis of *ippA1* and *ippA2* in strain D-6, the *ippR* knockout strain D-6*ΔippR*, and the *ippR*-complementary strain D-6*ΔippR/ippR* in the presence of 1.04 mM glucose or 1.04 mM IPP. The transcriptional level of the 16S rRNA gene was used as an internal standard, and the data in each column were calculated by the 2^−ΔΔ*CT*^ threshold cycle (*C_T_*) method using three replicates. (**D**) PCR amplification of the *ippA1A2* cluster using DNA (D), total RNA (R), and cDNA (cD) as the templates. The amplified products were detected by electrophoresis. (**E**) DNA elements in the promoter of *IPP1* operon. The IppR-binding site in the promoter region of *IPP1* operon is indicated by a gray box. Fragments F*_IPP1_* and P*_IPP1_* were underlined.

**Fig 2:**
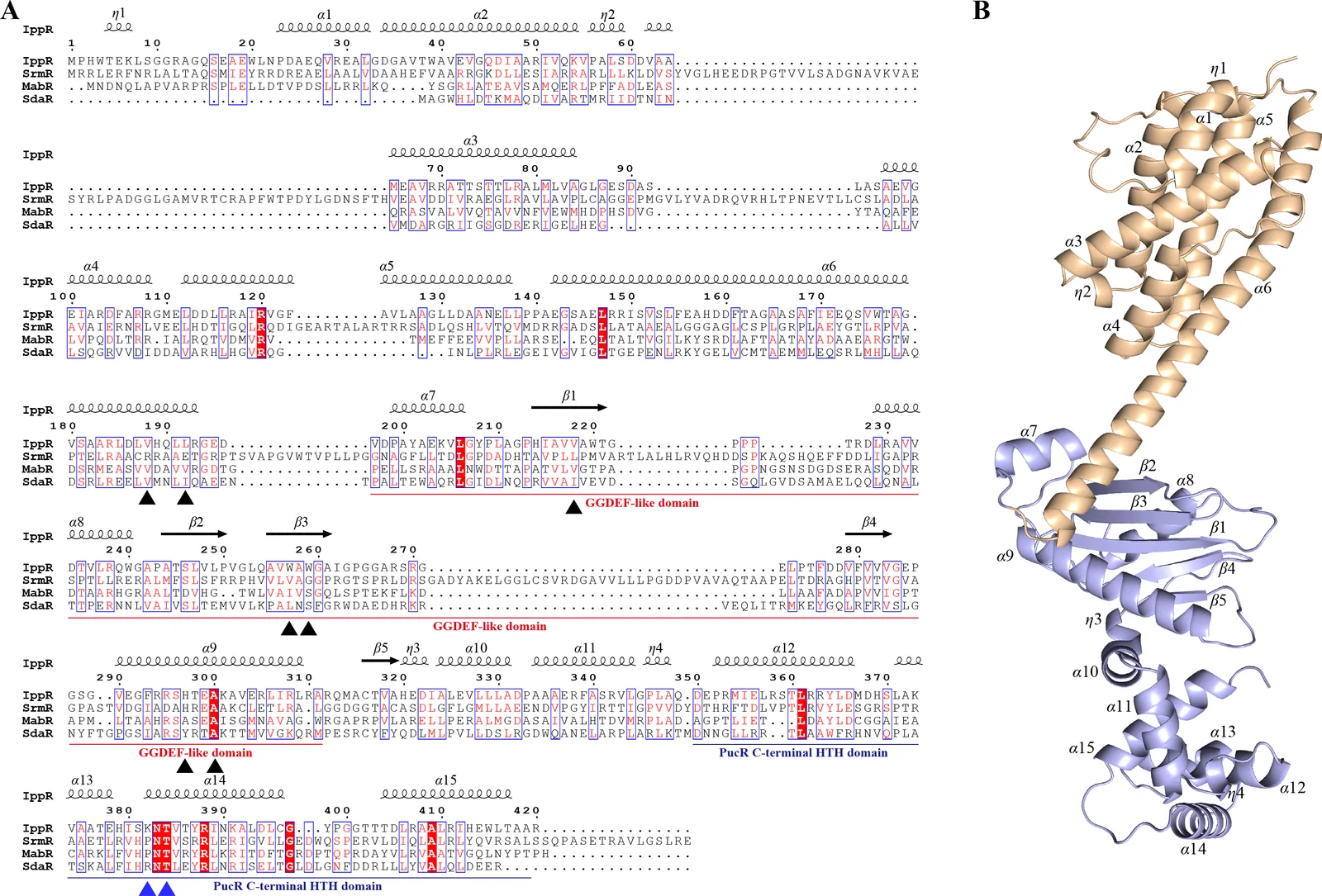
(**A**) Alignment of IppR with the PucR-type transcriptional regulator. IppR was aligned with SrmR (CAA45051), MabR (P9WPH5), and SdaR (P37047) using ClustalX. The GGDEF-like domain and the PucR C-terminal helix-turn-helix (HTH) domain were underlined. The key amino acids of IppR for binding to IPP and the promoter region of the *IPP1* operon were indicated by a black arrow and a blue arrow, respectively. (**B**) Structure of IppR predicted by ColabFold. The N-terminal domain was shown in wheat color, and the C-terminal domain was shown in light blue color.

Genome analysis of strain D-6 revealed a putative transcriptional regulator gene, *ippR*, upstream of the *ippA1A2* genes (Fig. 1B and Fig. S1). BLASTP analysis conducted in the NCBI non-redundant database indicated that IppR shared the highest sequence similarity (80.53%) with an uncharacterized PucR family regulator from *Rhodococcus aetherivorans* (WP_387433404), with 99% coverage. Phylogenetic analysis further demonstrated that IppR clustered with SrmR, MabR, and SdaR within a subclade of the PucR family (Fig. S1). Among functionally characterized PucR-type regulators, IppR showed the highest amino acid sequence identity to SrmR (29.66%), a regulator of spiramycin biosynthesis in *Streptomyces ambofaciens* BES2281 (17, 18). IppR shared 27.54% sequence identity with MabR, which regulates mycolic acid biosynthesis in *Mycobacterium tuberculosis* H37Rv (19, 20), and 27.66% identity with SdaR, which regulates the metabolism of d-galactarate, d-glycerate, and d-glucarate in *Escherichia coli* K12 (21). Multiple sequence alignment revealed that IppR contains a GGDEF-like domain (Val^197^-Ala^311^) and a PucR-type C-HTH domain (Asp^350^-Ala^419^), both of which are characteristic features of PucR family transcriptional regulators (Fig. 2A). Collectively, these results identify IppR as a new member of the PucR-type regulator.

To investigate the role of IppR in regulating the expression of the gene cluster, the *ippR* knockout strain D-6*ΔippR* and its complementary strain D-6*ΔippR/ippR* were constructed. Cells of strains D-6, D-6*ΔippR*, and D-6*ΔippR/ippR* were incubated in MSM supplemented with 1.04 mM IPP as the sole growth substrate. As shown in Fig. S2A, the wild-type strain D-6 exhibited a 6-h lag phase and completely degraded 1.04 mM IPP within 21 h, whereas strain D-6*ΔippR* lost the ability to degrade IPP. Additionally, the cell density of strain D-6 cultured with 1.04 mM IPP increased significantly from 3.0 × 10^6^ CFU/mL to 7.1 × 10^7^ CFU/mL (Fig. S2B), indicating that IPP served as the carbon and energy source for cell growth of strain D-6. In contrast, the cell density of strain D-6*ΔippR* cultured with IPP showed no significant change (Fig. S2B). Moreover, the degradation and growth characteristics of the complementary strain D-6*ΔippR/ippR* were comparable to those of the wild-type strain D-6 (Fig. S2).

The transcriptional levels of *ippA1* and *ippA2* were evaluated in strains D-6, D-6*ΔippR*, and D-6*ΔippR/ippR* grown on IPP or glucose using qRT-PCR. In strain D-6, the transcription levels of *ippA1* and *ippA2* in the presence of IPP were 25- and 27-fold higher, respectively, than those in the presence of glucose (Fig. 1C). However, no significant differences in transcriptional levels of these genes were observed in the *ippR* knockout strain D-6*ΔippR* between IPP- and glucose-grown cells (Fig. 1C). In the *ippR* complementary strain D-6*ΔippR/ippR*, the transcriptional levels of these genes were similar to those of wild-type strain D-6 (Fig. 1C). These results indicate that IppR functions as a transcriptional activator of the *ippA1A2* cluster.

### Organization and transcriptional analysis of the *ippA1A2* cluster

To investigate the transcript of the *ippA1A2* cluster, total DNA, RNA, and cDNA extracted from IPP-induced cells of strain D-6 were used as templates for PCR amplification. The PCR products were verified by gel electrophoresis. Among the four amplified fragments, only fragments F2 and F3 were amplified from cDNA (Fig. 1D). These results indicate that *ippA1*, *orf2560*, and *ippA2* are transcribed as a single operon, designated the *IPP1* operon.

To identify the promoter region of the *IPP1* operon, its TSS was determined by 5′-RACE using total RNA extracted from IPP-induced cells of strain D-6. The TSS of the *IPP1* operon was “A” at 97 bp upstream of the *ippA2* start codon (Fig. S3). The promoter region of *IPP1* operon, a 128-bp fragment located upstream of TSS, was designated as fragment P*_IPP1_* (Fig. 1E). A detailed analysis of this promoter region is shown in Fig. 1E.

### IPP as an effector that enhances IppR binding to the promoter region of the *IPP1* operon

The *ippR* was cloned from strain D-6 and expressed in *E. coli* BL21(DE3). Recombinant IppR was purified as a C-terminal 6×His-tagged protein using an Ni-NTA agarose resin matrix. The monomeric molecular weight of IppR was determined to be approximately 45.0 kDa by SDS-PAGE, consistent with its expected molecular weight (Fig. S4A). As shown in Fig. S5, gel filtration chromatography revealed that native IppR eluted as a dimer (the calculation method is described in the legend of Fig. S5). Collectively, these results demonstrate that IppR exists as a homodimer *in vitro*.

**Fig 3:**
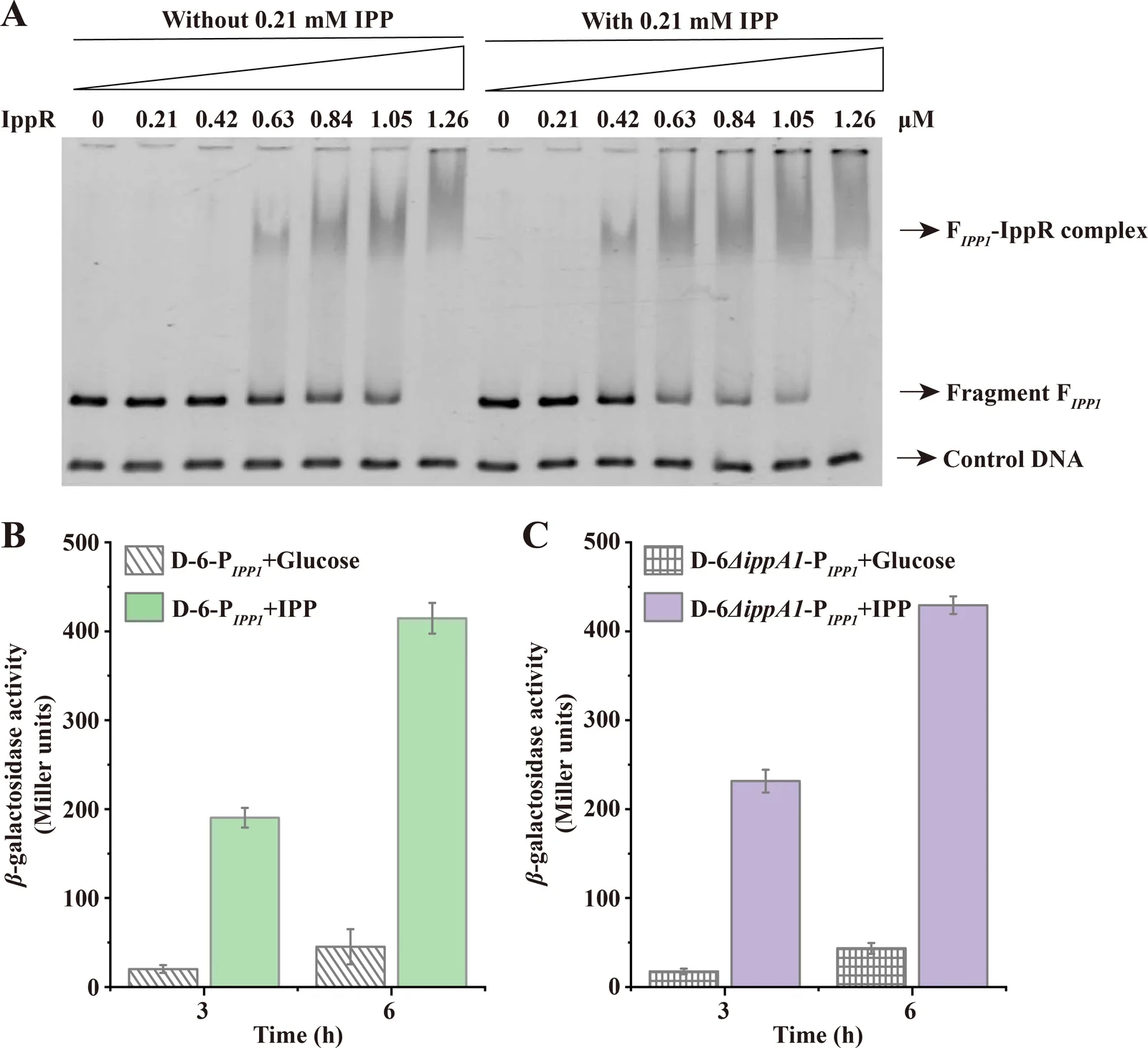
Determination of the effector of IppR. (**A**) Effects of IPP on the binding of IppR to the promoter region of *IPP1* operon (fragment F*_IPP1_*). EMSAs with fragment F*_IPP1_* (40 nM) and purified IppR (0–1.26 μM) without or with 0.21 mM IPP. The control DNA was a 125-bp fragment that was amplified from the *ippA1* gene. (**B**) The *β*-galactosidase activity assay was performed with strain D-6 carrying the P*_IPP1_-lacZ* transcriptional fusion, grown in the presence of 1.04 mM glucose or IPP. (**C**) The *β*-galactosidase activity assay was performed with strain D-6*ΔippA1* carrying the P*_IPP1_-lacZ* transcriptional fusion, grown in the presence of 1.04 mM glucose and IPP.

EMSA was performed to determine whether IppR binds to the promoter region of the *IPP1* operon (fragment F*_IPP1_*, the 225-bp fragment spanning from −128 to +97 of the TSS). The results showed that IppR bound to fragment F*_IPP1_* along with increasing amounts (0–1.26 μM) of IppR, with a detectable F*_IPP1_*-IppR complex forming at 0.63 μM IppR (Fig. 3A). In contrast, no DNA-protein complex was observed with a nonspecific control DNA fragment derived from *ippA1*, confirming the binding specificity between IppR and fragment F*_IPP1_* (Fig. 3A). Subsequently, the effect of IPP on the binding of IppR to fragment F*_IPP1_* was evaluated by EMSA. When IPP was present in the incubation mixture of IppR and fragment F*_IPP1_*, the binding capacity of IppR to the promoter was enhanced. As shown in Fig. 3A, the formation of the F*_IPP1_*-IppR complex required only 0.42 μM IppR in the presence of IPP, while 0.63 μM IppR was required in its absence. Additionally, at identical IppR concentrations (0.63, 0.84, and 1.05 μM), IPP treatment lowered the amount of free fragment F*_IPP1_* and promoted the formation of the F*_IPP1_*-IppR complex (Fig. 3A). These results indicate that IppR binds to the promoter region of the *IPP1* operon, and IPP is an effector to enhance this interaction.

To further investigate the effect of IPP on the binding of IppR to the promoter region of the *IPP1* operon, the promoter region was cloned into the *β*-galactosidase reporter plasmid pME6522 (27), generating the plasmid pME6522-P*_IPP1_*. The resulting promoter-*lacZ* fusion plasmid pME6522-P*_IPP1_* was subsequently introduced into strain D-6, generating derivative strain D-6-P*_IPP1_. β*-Galactosidase activity in strain D-6-P*_IPP1_* was induced using glucose and IPP. As illustrated in Fig. 3B, strain D-6-P*_IPP1_* exhibited low *β*-galactosidase activity (45 Miller units) in the presence of glucose, whereas IPP significantly increased its activity to 414 Miller units. These results suggest that IPP acts as the effector molecule of IppR. To validate this, the plasmid pME6522-P*_IPP1_* was introduced into the *ippA1* knockout strain D-6*ΔippA1*, which is unable to degrade IPP (16). As shown in Fig. 3C, IPP still significantly induced *β*-galactosidase activity in strains D-6*ΔippA1*-P*_IPP1_*. Additionally, IPC, 2-isopropylhydroquinone, and its downstream metabolites exhibit no effector activity toward IppR (data shown in the supplemental text). These results confirm that IPP functions as the effector molecule of IppR and activates transcription of the *ippA1A2* cluster.

### Key amino acids of IppR involved in IPP binding

To identify the amino acids in IppR required for IPP binding, molecular docking analyses of IPP to IppR were performed. Five structural models of IppR were predicted using the ColaFold platform based on the AlphaFold2 algorithm (Fig. S6). The reliability of these models was evaluated primarily using the mean predicted local distance difference test (pLDDT) scores, together with predicted aligned error (PAE) profiles (Fig. S6). Among the five models, model IV showed the highest mean pLDDT score (88.0), compared with models I (87.1), II (85.5), III (87.6), and V (85.2) (Fig. S6). Therefore, model IV was selected for subsequent structural analysis. The predicted structure of the IppR monomer consists of two distinct regions: an N-terminal domain (N-IppR, Met^1^ to Glu^195^) and a C-terminal domain (C-IppR, Asp^196^ to Arg^420^) (Fig. 2), consistent with the domain organization of *Mycobacterium tuberculosis* MabR (PDB IDs: 9F81 and 9F80), the only structurally characterized PucR-type regulator reported to date (23). For the N-terminal domain, both IppR and MabR adopt an all-*α* structure. Specifically, the predicted N-IppR is composed of two 3_10_-helices (*η*1 and *η*2) and six *α*-helices (*α*1 to *α*6), while N-MabR consists of six *α*-helices. For the C-terminal domain, both IppR and MabR possess a GGDEF-like domain and a PucR-type C-HTH domain. Specifically, the GGDEF-like domain of C-IppR (Val^197^-Ala^311^) comprises three *α*-helices (*α*7 to *α*9) and four *β*-strands (*β*1 to *β*4), while the corresponding domain in MabR contains three 3_10_-helices, five *α*-helices, and five *β*-strands. The PucR-type C-HTH domain of IppR (Asp^350^-Ala^419^) contains four *α*-helices (*α*12 to *α*15), similar to MabR, which also adopts four *α*-helices. In addition, the two domains of IppR are connected by two 3_10_-helices (*η*3 and *η*4), two *α*-helices (*α*10 and *α*11), and one *β*-strand (*β*5), while only two α-helices mediate the interdomain connection in MabR.

**Fig 4:**
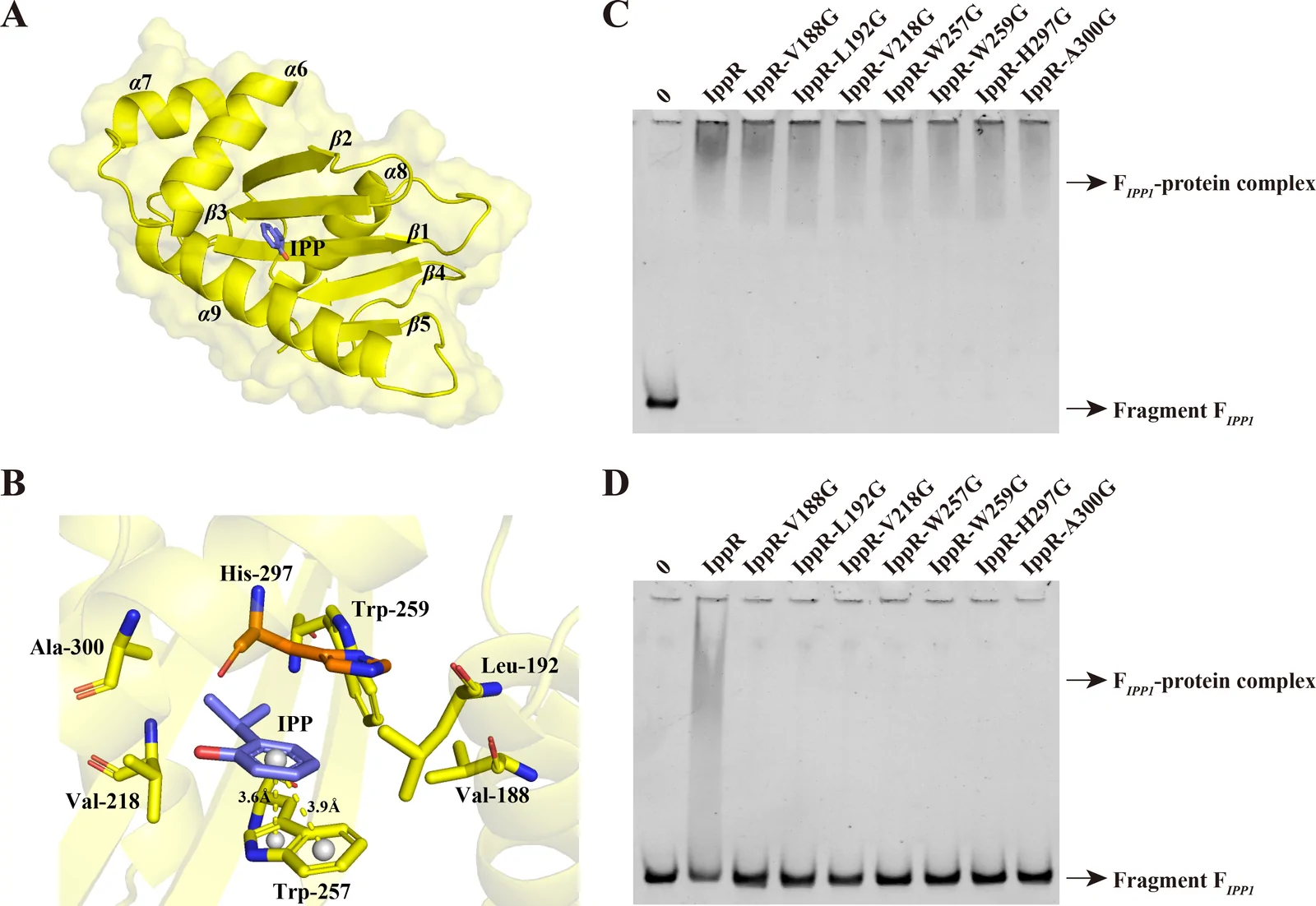
Identification of key amino acids for IPP binding in IppR. (**A**) The IPP-binding domain of IppR. (**B**) Residues in the IPP-binding pocket of IppR. Residues in yellow are hydrophobic residues, residues in orange are amphipathic residues, and the molecule in blue is IPP. π-π stacking interaction are shown in yellow dotted lines. (**C**) EMSAs using fragment F*_IPP1_* (40 nM) with IppR and its mutant proteins (1.26 μM). (**D**) EMSAs using fragment F*_IPP1_* (40 nM) with IppR and its mutant proteins (0.42 μM) in the presence of 0.21 mM IPP.

To predict the interaction between IPP and IppR, molecular docking of IPP was performed against the putative active sites of IppR using the AutoDock program. The results showed that the substrate binding pocket of IPP was located within the GGDEF-like domain and the adjacent region. The IPP binding pocket was comprised of five antiparallel *β*-strands (*β*1 to *β*5) surrounded by four *α*-helices (*α*6 to *α*9) (Fig. 4A). Visualization analysis revealed that the vicinity of IPP contained several hydrophobic residues, including Val188, Leu192, Val218, Trp257, Trp259, and Ala300, as well as the amphipathic residue His297 (Fig. 4B). Val188, Trp257, Ala300, and the amphipathic His297 engage in hydrophobic interactions with IPP. These residues encircle IPP, creating a hydrophobic microenvironment in the predicted effector-binding site (Fig. 4B). The parallel orientation of the IPP benzene ring relative to the aromatic ring of Trp257 enables strong π-π stacking interactions (Fig. 4B), which likely stabilize IPP within the IppR binding pocket and enhance its binding affinity.

To assess the role of these residues in IPP binding, Val188, Leu192, Val218, Trp257, Trp259, His297, and Ala300 were individually substituted with glycine, generating mutants IppR-V188G, IppR-L192G, IppR-V218G, IppR-W257G, IppR-W259G, IppR-H297G, and IppR-A300G (Fg. S4B). These mutant proteins could still bind to the fragment F*_IPP1_* when their concentration reached 1.26 μM (Fig. 4C). However, the fragment F*_IPP1_* was not shifted when incubated with the mutant proteins (0.42 μM) in the presence of 0.21 mM IPP, while the F*_IPP1_*-IppR complex formed under the same conditions (Fig. 4D). These findings indicate that the mutant proteins maintained DNA-binding capability to fragment F*_IPP1_* but lost responsiveness to IPP. Moreover, strains D-6*ΔippR/ippR*-V188G, D-6*ΔippR/ippR*-L192G, D-6*ΔippR/ippR*-V218G, D-6*ΔippR/ippR*-W257G, D-6*ΔippR/ippR*-W259G, D-6*ΔippR/ippR*-H297G, and D-6*ΔippR/ippR*-A300G were unable to degrade IPP (Table S1). These results are consistent with Val188, Leu192, Val218, Trp257, Trp259, His297, and Ala300 playing key roles in IPP binding.

### Binding sites of IppR to the promoter region of the *IPP1* operon

**Fig 5:**
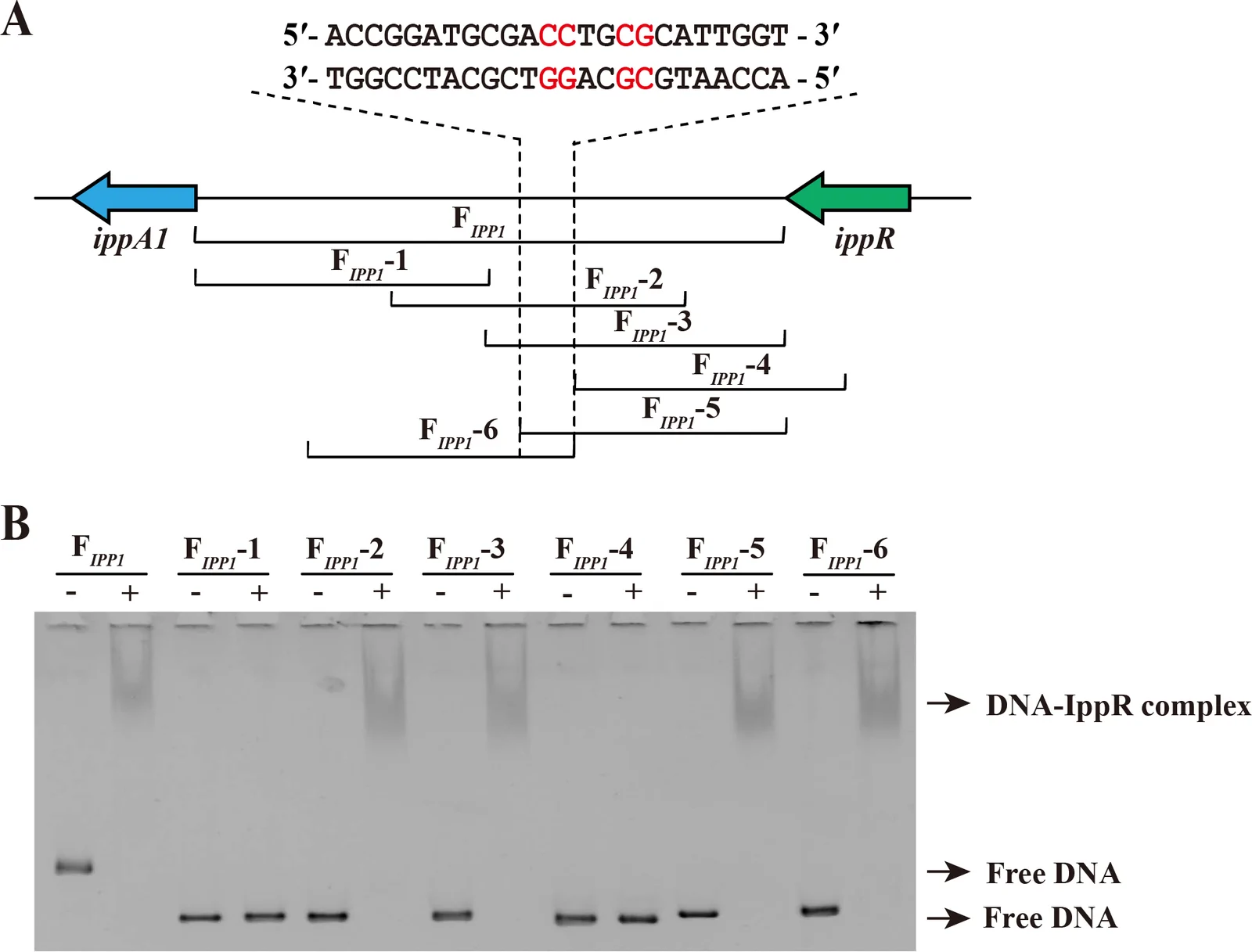
Identification of IppR-binding site within the promoter regions of fragments F*_IPP1_*. (**A**) Schematic diagram of fragment F*_IPP1_* and its sub-fragments F*_IPP1_*-1 to F*_IPP1_*-6 used to determine the IppR-binding site. The locations of the fragments are shown below. The nucleotide sequence above the fragments shows the IppR-binding site within the promoter regions of fragment F*_IPP1_*, and the CC/CG box was highlighted in red. (**B**) EMSA of fragment F*_IPP1_* and sub-fragments F*_IPP1_*-1 to F*_IPP1_*-6 in panel A with IppR. The lanes contain the following: the DNA fragment (40 nM) alone (−) and the DNA fragment (40 nM) with 1.26 μM IppR (+).

To determine the key binding sites of IppR, the fragment F*_IPP1_* was divided into six sub-fragments F*_IPP1_*-1 to F*_IPP1_*-6 and analyzed by EMSA (Fig. 5A). As shown in Fig. 5B, fragment F*_IPP1_* and sub-fragments F*_IPP1_*-2, F*_IPP1_*-3, F*_IPP1_*-5, and F*_IPP1_*-6 were shifted upon incubation with IppR, forming the DNA-IppR complex. These five fragments shared a 24-bp overlapping DNA motif 5′-ACCGGATGCGACCTGCGCATTGGT-3′.

**Fig 6:**
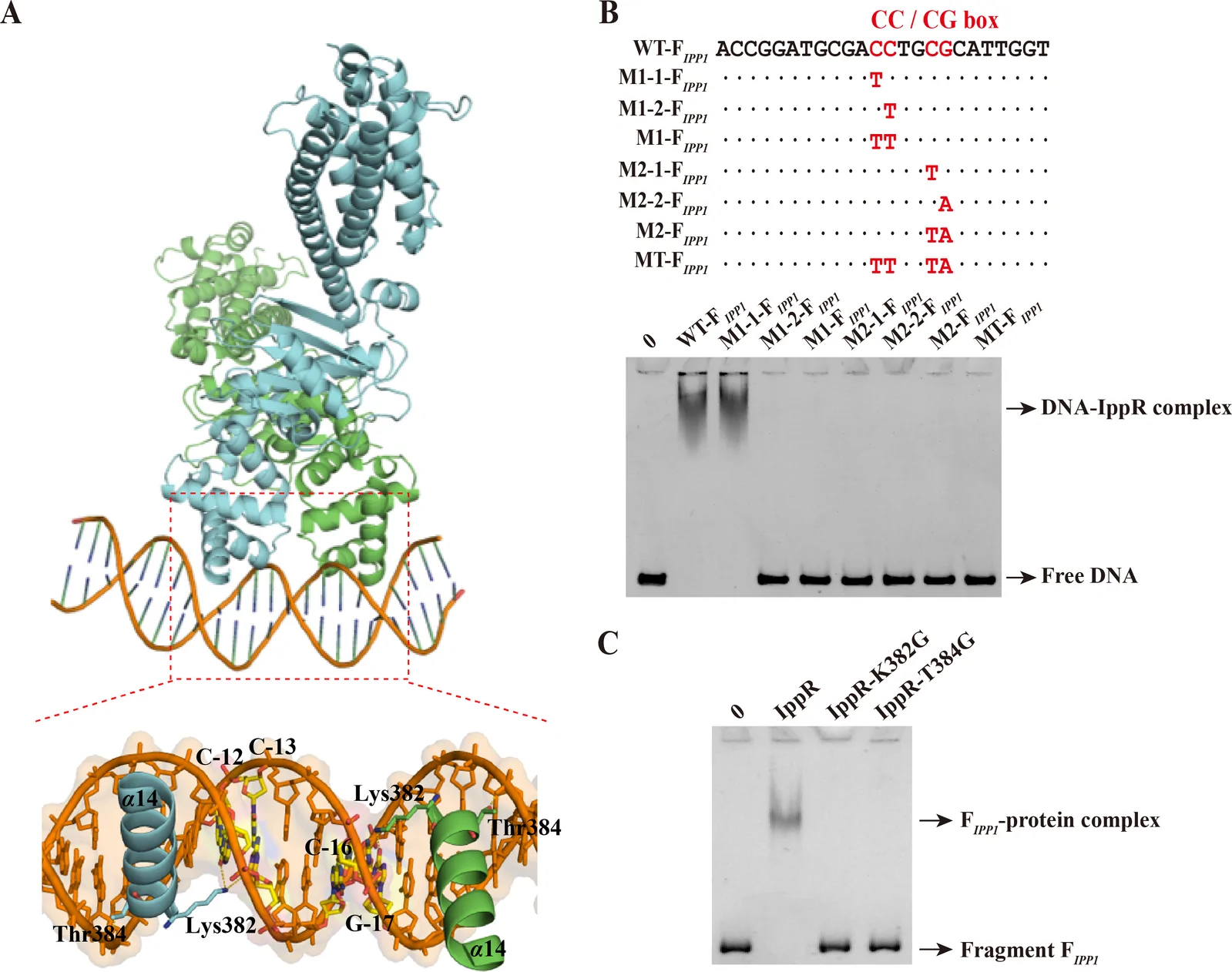
(**A**) Structure model of the DNA-IppR complex. The IppR dimer is bound to the 24-bp DNA motif. The two monomers of IppR are represented in light blue and light green, respectively. The close-up view shows IppR bound to DNA, with the interacting bases in yellow and the predicted contact residues Lys382 and Thr384 as sticks. (**B**) Diagram of the wild-type sequence of fragment F*_IPP1_* and its mutant sequences was shown at the top. The mutant nucleotides between the wild-type sequence and the mutant type sequences are shown in red. Lane 0, the wild-type promoter fragment F*_IPP1_* (40 nM) alone, lanes WT-F*_IPP1_* to MT-F*_IPP1_*, the mutant DNA fragment (40 nM) with purified IppR (1.26 μM). (**C**) EMSAs using fragment F*_IPP1_* (40 nM) with purified IppR and its mutant proteins (1.26 μM).

To further investigate essential binding sites within the 24-bp motif, molecular docking with the target motif to IppR dimer was performed using the HDOCK Server (28, 29). The predicted DNA-IppR complex structure indicated that the IppR dimer recognizes the 24-bp DNA motif through the C-HTH domain of each monomer. As shown in Fig. 6A, the *α*14 of IppR (the third *α-*helix within the C-HTH domain of each IppR monomer) acts as the recognition helix and inserts into the DNA major groove. In the complex, Lys382 forms hydrogen bonds with C-12, C-13, C-16, and G-17 (Fig. 6A). To investigate the role of the CC/CG box in IppR binding, various mutant DNA fragments of F*_IPP1_* were analyzed using EMSAs. As shown in Fig. 6B, only the M1-1-F*_IPP1_* fragment could bind to IppR to form DNA-IppR complex, while all other mutant fragments lost binding activity. These results further suggest the critical role of the CC/CG box in IppR sequence recognition.

### Key amino acids of IppR involved in binding to the promoter DNA

In addition to Lys382, which forms hydrogen bonds with the CC/CG box, Thr384 also forms non-specific hydrogen bonds with the phosphate backbone of the 24-bp DNA motif (Fig. 6A). To determine the roles of Lys382 and Thr384 for DNA binding in IppR, two variants (IppR-K382G and IppR-T384G) were constructed (Fig. S4C). As shown in Fig. 6C, the DNA-binding abilities of both variants were significantly altered, resulting in a complete disruption of fragment F*_IPP1_*-binding capacity. Furthermore, strains D-6*ΔippR/ippR*-K382G and D-6*ΔippR/ippR*-T384G lost the ability to degrade IPP (Table S1). These results are consistent with Lys382 and Thr384 playing crucial roles in the DNA-binding function of IppR.

## DISCUSSION

This study presents evidence that IppR is a PucR-type transcriptional activator that positively regulates the transcription of the *ippA1A2* genes, which encode an IPP hydroxylase. This role is consistent with most characterized PucR-type regulators, which act as transcriptional activators (17–26). A notable exception is PucR itself, which acts as a dual regulator with both an activator and a repressor activity. Based on the presented data, we propose that IppR binds IPP as an effector and functions similarly as the PucR-type activators. However, further studies are required to elucidate the mechanism of action of PucR family transcriptional regulators.

The GGDEF-like domain shares evolutionary similarity with the canonical GGDEF domain, which plays a pivotal role in binding the substrate guanosine triphosphate in diguanylate cyclase (30–32). Structurally, the GGDEF domain of diguanylate cyclase adopts a typical fold consisting of five antiparallel *β*-strands surrounded by five peripheral *α*-helices. The structure of the GGDEF-like domain of IppR comprises four anti-parallel *β*-strands surrounded by three *α*-helices. Despite differences in secondary structural element composition, the two domains exhibit a conserved overall topology characterized by a central *β*-sheet wrapped by peripheral *α*-helices. Megalizzi et al. hypothesized that the GGDEF-like domain of PucR-type regulators could potentially function as the effector-binding site (23). However, this hypothesis has not been experimentally verified. In this study, homology modeling combined with molecular docking analysis identified that the GGDEF-like domain of IppR, along with its neighboring amino acid residues, collectively form the substrate-binding pocket for IPP (Fig. 4A). The IppR binding pocket forms a hydrophobic microenvironment consistent with the hydrophobic characteristics of IPP. Hydrophobic interactions with IPP and π-π stacking interaction mediated by Trp257 enhance the stabilization of IPP within the active site (Fig. 4B). Collectively, these results confirm that the GGDEF-like domain and its vicinity are the essential binding centers for the effector molecule IPP. This study offers direct evidence that the GGDEF-like domain of PucR family regulators is directly involved in effector binding and functional regulation, representing the first report of the GGDEF-like domain exerting regulatory effects through effector binding.

Based on previous reports, PucR-type regulatory proteins exhibit diverse target binding sites on promoter DNA. Beier et al. found that PucR, a regulatory protein controlling the purine catabolic pathway in *Bacillus subtilis* 168, recognizes a conserved binding motif on promoter DNA (24). In contrast, other characterized PucR-type members bind promoter DNA via recognition of inverted repeat sequences, including AdeR (25), PrcR (26), MabR (19), and GabR (22). AdeR binds the promoter of L-alanine dehydrogenase-encoding *ald* to regulate gene transcription (25). PrcR regulates proline utilization in strain 168 by binding to the promoter DNA of the *putBCP* operon (26). In *Mycobacterium tuberculosis* H37Rv, MabR controls mycolic acid biosynthesis (19). GabR in *Corynebacterium glutamicum* ATCC 13032 regulates 4-aminobutyric acid metabolism (22). Additionally, no target DNA-binding sites have been reported for SrmR and SdaR, two members of the PucR family. In this study, the key promoter region bound by IppR was identified as 5′- ACCGGATGCGACCTGCGCATTGGT-3′ (Fig. 5). Molecular docking and site-directed mutagenesis experiments validated that mutation of the CC/CG box within this region eliminated the retardation of the DNA fragment by IppR (Fig. 6A and B), indicating that the CC/CG box is essential for stable formation of the DNA-IppR complex. This binding site differs from previously reported PucR family binding patterns, providing a novel motif type for promoter recognition by PucR family transcriptional regulators.

The HTH domain is widespread among diverse regulators and mainly functions in recognizing and binding promoter DNA of target genes (33–40). Members of the PucR family harbor a C-terminal HTH domain that is critical for DNA recognition (23). Consistent with these observations, our structural characterization identifies that IppR contains a PucR-type HTH domain at its C-terminal. Structural analysis of the predicted IppR-DNA complex demonstrates that the third *α*-helix within this domain inserts into the DNA major groove, thereby mediating promoter DNA binding (Fig. 6B). In comparison, structural analysis of *Mycobacterium tuberculosis* MabR, the sole structurally characterized PucR-type regulator, has confirmed that its C-terminal HTH domain uses the second *α*-helix to insert into the DNA major groove for sequence-specific binding (23). Lys382 of IppR is predicted to form hydrogen bonds with the CC/CG box, while Thr384 forms non-specific hydrogen bonds with the phosphate backbone of the 24-bp core DNA-binding motif (Fig. 6A). These two residues are predicted to be essential for IppR recognition and binding to the promoter DNA of the *IPP1* operon. Notably, although several PucR-family regulators have been functionally characterized, the precise PucR-type regulators’ DNA binding mode and the critical residues mediating their interaction have not been defined to date. Our findings not only provide a basis for clarifying the molecular mechanism of DNA-IppR binding but also confirm that the HTH domain is the key functional domain responsible for DNA recognition and binding in PucR family regulators.

## MATERIALS AND METHODS

### Chemicals and media

2-Isopropylphenol (IPP; 98.0% purity) was purchased from Macklin Biochemical Co., Ltd. (Shanghai, China). Luria-Bertani (LB, pH 7.0) medium consisted of the following components (in g liter^−1^): 10.0 tryptone, 5.0 yeast extract, and 10.0 NaCl. Mineral salt medium (MSM, pH 7.0) consisted of the following components (in g liter^−1^): 1.5 K_2_HPO_4_, 0.5 KH_2_PO_4_, 0.2 MgSO_4_·7H_2_O, 1.0 NH_4_NO_3_, and 1.0 NaCl. IPP (1.04 mM) or glucose (1.04 mM) was used as the sole growth substrate for cell growth of strain D-6 and its related mutant strains unless stated otherwise.

### Bacterial strains, plasmids, and culture conditions

All bacterial strains and plasmids utilized in this study were listed in Table S2, and the primers were listed in Table S3. *Escherichia coli* strains were cultivated at 37°C in LB medium. *Rhodococcus* sp. D-6, an isoprocarb (IPC)-degrading strain utilizing IPC as the sole carbon and energy source for its growth, and strain D-6*∆ippA1*, an *ippA1* knockout strain that lost its IPP degradation ability, were previously isolated and constructed by our lab (11, 16). Strain D-6 and its derivatives were grown at 30°C in LB medium. Antibiotics were supplemented according to Table S2. Streptomycin (Str), tetracycline (Tc), and kanamycin (Km) were supplemented to final concentrations of 25 μg mL^−1^, 15 μg mL^−1^, and 50 μg mL^−1^, respectively.

### Genetic manipulations

The *ippR* gene was knocked out through a double-crossover event. Two DNA fragments, corresponding to the 1,300 bp upstream and downstream flanking regions of the *ippR* gene, were amplified using primer pairs *ippR*-upF/*ippR*-upR and *ippR*-downF/*ippR*-downR (listed in Table S3), respectively. These two PCR fragments were combined through overlap extension PCR and subsequently cloned into *Bam*HI- and *Eco*RI-digested pK18*mobSacB* using the ClonExpress II one-step cloning kit (Vazyme, Nanjing, China), resulting in the generation of pK18-*ippR* (41). Subsequently, pK18-*ippR* was introduced into strain D-6 via electroporation. Single-crossover mutants were screened on LB plates supplemented with Km (50 μg mL^−1^) and Str (25 μg mL^−1^) and confirmed through PCR amplification. Double-crossover mutants were selected on LB plates containing Str (25 μg mL^−1^) and 20% sucrose and verified by PCR amplification. The double-crossover knockout strain was sequenced to ensure accurate gene deletion and designated as D-6*ΔippR*.

To obtain the *ippR*-complementary strain D-6*ΔippR/ippR*, the *ippR* gene was amplified with the primer pair *ippR*-hbF/*ippR*-hbR (listed in Table S3) from genomic DNA of strain D-6. The resulting PCR product was ligated into pRESQ, generating pRE-*ippR*, which was then electroporated into strain D-6*∆ippR* for complementation (42).

The construction of point mutant strains was similar to the procedure used for *ippR* complementation, with the differences described below. Primers carrying desired mutation sites were used for fragment amplification. The resulting plasmids were electroporated into strain D-6*ΔippR* to generate corresponding complementary strains.

### Growth and degradation assay

Strain D-6 and its derivates were inoculated into LB medium, harvested by centrifugation, and washed three times with MSM. The washed cells were then resuspended in MSM containing 1.04 mM IPP, with an initial density of approximately 3.0 × 10^6^ CFU/mL. The residual concentration of IPP was determined by HPLC analysis. Concurrently, the growth of these strains was quantified by enumerating viable colonies on LB agar plates supplemented with 25 mg/L Str. All experiments were conducted in triplicate.

### Quantitative reverse transcription-PCR

Strains D-6, D-6*ΔippR*, and D-6*ΔippR/ippR* were cultured to an optical density at 600 nm (OD_600_) of 0.6 in LB medium containing Str (25 μg mL^−1^). Cells were then harvested by centrifugation (6,000 × *g* for 5 min), washed three times with fresh MSM, and resuspended in MSM to a final OD_600_ of 1.0 at 30°C in the presence of 1.04 mM IPP. MSM containing 1.04 mM glucose was used as the negative control. Upon reaching 40% to 60% of IPP degradation, total RNA was extracted using an RNA isolation kit (TaKaRa, Dalian, China). cDNA was synthesized using the PrimeScript RT reagent kit (Vazyme, Nanjing, China) and subsequently used as a template for quantitative PCR (qPCR). The 16S rRNA gene served as an internal standard control, and relative gene expression was calculated based on the 2^−ΔΔ*CT*^ threshold cycle (*C_T_*) method.

### Determination of the transcription start sites

Total RNA was extracted from strain D-6 cultured in MSM supplemented with 1.04 mM IPP using an RNA isolation kit (TaKaRa, Dalian, China). The transcription start site (TSS) of the *IPP1* operon, including genes *ippA1*, *orf2560*, and *ippA2*, was identified using 5′-rapid amplification of cDNA ends (5′-RACE) (Sangon, Shanghai, China). First-strand cDNA was reverse transcribed using primer TSS-A-1 (listed in Table S3), followed by cDNA tailing with terminal transferase and dGTP. The dG-tailed cDNA was then amplified using the abridged anchor primer (APP) and TSS-A-2 (listed in Table S3). The resulting amplification product underwent a nested PCR with AAP and primer TSS-A-3 (listed in Table S3) to generate the final DNA fragment. The final PCR product was cloned into the pMD19-T vector (TaKaRa, Japan) and sequenced using M13F-47 and M13R-48 primers. The promoter region of the *IPP1* operon was predicted using BDGP (43) and BacPP (44).

### Protein expression and purification

The *ippR* gene was amplified from strain D-6 using the primer pair *ippR*-F/*ippR*-R (listed in Table S3) and cloned into pET-29a(+) to yield pET-*ippR* (45, 46). The plasmid pET-*ippR* was transformed into *E. coli* BL21(DE3), and the cells were cultured at 37°C in LB medium with 50 µg mL^−1^ Km until OD_600_ reached approximately 0.6. Subsequently, cells were induced at 16°C for 10 h by adding 0.1 mM isopropyl-*β*-d-thiogalactopyranoside. After induction, cells were harvested, washed, and lysed by sonication. Subsequently, the C-terminal 6×His-tagged protein IppR was purified using an Ni-NTA agarose resin matrix. The concentration and molecular weight of IppR were determined by the Bradford method (47) and sodium dodecyl sulfate-polyacrylamide gel electrophoresis (SDS-PAGE), respectively.

The multimeric state of native IppR was determined by gel filtration chromatography (48). IppR was loaded onto a Superdex 200 Increase 10/300 GL column (GE Healthcare) with 20 mM Tris-HCl buffer (pH 7.4, containing 0.1 M NaCl) as the mobile phase (0.4 mL min^−1^) at 25°C. Standard proteins, including *β*-galactosidase (116 kDa), phosphorylase b (97 kDa), bovine serum albumin (66 kDa), and ovalbumin (45 kDa), were used. The molecular weight (MW) of IppR was calculated from the linear regression of the standard curve, which was generated by plotting the logarithm of the molecular weights of the standards against their corresponding retention volume (*V*), following the equation Log_10_(Mw) = *a* × *V* + *b,* where *a* is the slope and *b* is the intercept of the calibration curve.

The construction, expression, and purification of mutant IppR proteins were performed similarly to those of wild-type IppR, with the differences described below. Mutants of the *ippR* gene were constructed using overlap PCR described by Zhu et al. (49).

### Electrophoretic mobility shift assay

The promoter regions of the *IPP1* operon were amplified from the genomic DNA of strain D-6 using the primer pair F*_IPP1_*-F/F*_IPP1_*-R presented in Table S3. A 125-bp region of *ippA1* used as the negative control was amplified using the primer pair *ippA1*-F/*ippA1*-R (Table S3). To explore the core binding sites of IppR, subfragments F*_IPP1_*-1 to F*_IPP1_*-6 of the promoter region of the *IPP1* operon were amplified using primers listed in Table S3. Point mutations in the promoter were generated via PCR, with the principle of mutating G and C to A and T, respectively. All mutated promoter DNA was amplified using primers listed in Table S3. The 40 nM promoter probe was incubated with purified IppR (0–1.26 µM) in EMSA binding buffer, which consisted of 20 mM Tris-HCl, 1 mM dithiothreitol, 0.5 mM EDTA, 50 mM KCl, and 5% (vol/vol) glycerol. To determine whether the effector of IppR was IPP, 0.21 mM IPP was added to the reaction system. The reaction mixtures were incubated at 25°C for 30 min and subsequently resolved on a 5% (vol/vol) native polyacrylamide gel by electrophoresis in 0.5 × Tris-glycine-EDTA buffer for 1 h. The gels were stained with SYBR green I for 15 min to visualize the DNA and DNA-protein complexes.

### Reporter plasmid construction and *β*-galactosidase activity assay

The promoter region of the *IPP1* operon was amplified using primer pair *ippA2*-6522-F and *ippA2*-6522-R listed in Table S3. The purified PCR product was digested with *Eco*RI and *Pst*I and ligated with pME6522 (27) to construct plasmid pME6522-P*_IPP1_*. Subsequently, plasmid pME6522-P*_IPP1_* was electroporated into strains D-6 and D-6*ΔippA* to yield strains D-6-P*_IPP1_* and D-6*ΔippA*-P*_IPP1_*, respectively.

*β*-Galactosidase activity was assayed using strain D-6-P*_IPP1_* grown in MSM supplemented with 1.04 mM IPP or 1.04 mM glucose, respectively. Cultures were incubated at 30°C with shaking at 180 rpm. *β*-Galactosidase activity assays utilized *o*-nitrophenyl-*β*-d-galactopyranoside (ONPG) as substrate, with 300 μL culture samples harvested for analysis. The observed activity was normalized to the optical density of the culture at 600 nm, the optical density of *o*-nitrophenol (ONP) at 420 nm, and expressed in Miller units (50). One Miller unit was defined as the amount of enzyme that catalyzed the conversion of ONPG into 1 µmol ONP per min.

### Structure modeling

The structural model of IppR was predicted using ColabFold (AlphaFold2.ipynb-Colaboratory), and the 3D conformer of IPP was obtained from PubChem (https://pubchem.ncbi.nlm.nih.gov/) (51, 52). IPP and the 24-bp motif were docked to IppR using AutoDock Vina software (53, 54) and HDOCK server (http://hdock.phys.hust.edu.cn/) (28, 29), respectively. Figures were generated using PyMOL software.

### Analytical techniques

To analyze IPP, 1 mL of degradation culture (MSM containing IPP inoculated with strain D-6 and its variant strains) was thoroughly mixed with 1 mL of methanol and centrifuged for 5 min at 12,000 × *g*. The supernatants were subsequently analyzed by HPLC (Thermo Fisher Scientific). The mobile phase consisted of methanol-water-glacial acetic acid (50:49:1, vol/vol/vol) at a flow rate of 1.0 mL min^−1^. Elution was monitored at 215 nm, with an injection volume of 20 μL and a column temperature of 40°C.

## Data Availability

The genome sequence of *Rhodococcus* sp. D-6 has been deposited in the GenBank database under the accession numbers CP132970 to CP132972 (11).

## SUPPLEMENTAL MATERIAL

Supplemental material aem.01277-26-s0001.pdf Tables S1 to S3, Fig. S1 to S6, and supplemental text.

ASM does not own the copyrights to Supplemental Material that may be linked to, or accessed through, an article. The authors have granted ASM a non-exclusive, world-wide license to publish the Supplemental Material files. Please contact the corresponding author directly for reuse.
