# Supplementary material for: The PucR-type activator IppR regulates 2-isopropylphenol hydroxylation in strain Rhodococcus sp. D-6
Source: Appl Environ Microbiol. 2026 Aug 6;92(9):e01277-26. doi: 10.1128/aem.01277-26 (PMC13599513; doi:10.1128/aem.01277-26)
Supplement: Supplementary file 1 [file aem.01277-26-s0001.pdf]

The PucR-type activator IppR regulates 2-isopropylphenol hydroxylation in strain *Rhodococcus* sp. D-6

Qian Zhu, [a] Kangning Wei, [a] Yike Lyu, [a] Qi Xie, [a] Kaihua Pan, [a] Qian Li, [a] Weihao Zhu, [a] Zhijian Ke, [b, c] Jiguo Qiu, [a] Mingliang Zhang, [a] Junqiang Hu, [a] Shang Dai, [a,#] Qing Hong, [a,#]

[a] Department of Microbiology, College of Life Sciences, Nanjing Agricultural University, Key Laboratory of Agricultural and Environmental Microbiology, Ministry of Agriculture and Rural Affairs, Nanjing 210095, PR China.

[b] College of Biological and Chemical Engineering, Jiaxing University, Jiaxing 314001, PR China.

[c] Research Center for Planetary Health, Jiaxing University, Jiaxing 314001, PR China.

[#] Author for correspondence: Shang Dai and Qing Hong

E-mail address: daishang@njau.edu.cn; hongqing@njau.edu.cn.

Tel: +86-25-84396685; Fax: +86-25-84395326

RUNNING TITLE: Transcriptional regulator IppR.

KEYWORDS: *Rhodococcus* sp. D-6, isoprocarb degradation, two-component 2-isopropylphenol monooxygenase gene *ippA1A2*, transcriptional regulation, PucR-type regulator

Table S1 Comparison of the relative activities of IPP degradation by strain D-6*Δ ippR/ippR* and its mutant strains.

Strains	Relative activity [a] (%)
D-6*ΔippR/ippR*	100 ± 2.4
D-6*ΔippR/ippR*-V188G	ND
D-6*ΔippR/ippR*-L192G	ND
D-6*ΔippR/ippR*-V218G	ND
D-6*ΔippR/ippR*-W257G	ND
D-6*ΔippR/ippR*-W259G	ND
D-6*ΔippR/ippR*-H297G	ND
D-6*ΔippR/ippR*-A300G	ND
D-6*ΔippR/ippR*-K382G	ND
D-6*ΔippR/ippR*-T384G	ND

[a] The IPP degradation activity of strain D-6*ΔippR/ippR* was defined as 100%, and the relative activity of its mutant strains was calculated. Results are the average of triplicate measurements ± standard deviation.

ND, not detectable.

Table S2 Strains and plasmids used in this study

Strain or plasmid	Characteristic(s)[a]	Source or reference(s)
E. coli strains		
DH5α	F^{-}*recA1 endA1 thi-1 supE44 relA1 deoRΔ(lacZYA-argF)U169 80dlacZΔM15*	TaKaRa
BL21 (DE3)	F^{-}*ompT hsdS*(r_B^{-} m_B^{-}) *gal dcm lacY1* (DE3)	TaKaRa
Rhodococcus sp. strains		
D-6	Wild type, able to degrade IPC and IPP; Str^{r}	This study
D-6Δ*ippA1*	*ippA1*-disrupted mutant from stain D-6; Str^{r}	This study
D-6Δ*ippR*	*ippR*-disrupted mutant from stain D-6; Str^{r}	This study
D-6Δ*ippR/ippR*	D-6Δ*ippR* harboring pRE-*ippR*; Str^{r}	This study
D-6Δ*ippR/ippR*-V188G	D-6Δ*ippR* harboring pRE-*ippR*-V188G; Str^{r}	This study
D-6Δ*ippR/ippR*-L192G	D-6Δ*ippR* harboring pRE-*ippR*-L192G; Str^{r}	This study
D-6Δ*ippR/ippR*-V218G	D-6Δ*ippR* harboring pRE-*ippR*-V218G; Str^{r}	This study
D-6Δ*ippR/ippR*-W257G	D-6Δ*ippR* harboring pRE-*ippR*-W257G; Str^{r}	This study
D-6Δ*ippR/ippR*-W259G	D-6Δ*ippR* harboring pRE-*ippR*-W259G; Str^{r}	This study
D-6Δ*ippR/ippR*-H297G	D-6Δ*ippR* harboring pRE-*ippR*-H297G; Str^{r}	This study
D-6Δ*ippR/ippR*-A300G	D-6Δ*ippR* harboring pRE-*ippR*-A300G; Str^{r}	This study
D-6Δ*ippR/ippR*-K382G	D-6Δ*ippR* harboring pRE-*ippR*-K382G; Str^{r}	This study
D-6Δ*ippR/ippR*-T384G	D-6Δ*ippR* harboring pRE-*ippR*-T384G; Str^{r}	This study
D-6-P_{IPP1}	D-6 containing plaimid pME6522-P_{IPP1}, Str^{r} Tc^{r}	This study
D-6Δ*ippA*-P_{IPP1}	D-6Δ*ippA* containing plaimid pME6522-P_{IPP1}, Str^{r} Tc^{r}	This study
Plasmids		
pK18*mobsacB*	Gene knockout vector; *ori*T, *sac*B, Km^{r}	(41)
pRESQ	Cloning vector; Km^{r}	(42)
pK18-*ippR*	*ippR* gene knockout vector containing upstream and downstream homologous regions of *ippR*; Km^{r}	This study
pRE-*ippR*	pRESQ harboring *ippR*; Km^{r}	This study

pRE-*ippR*-V188G	pRESQ harboring *ippR*-V188G; Str^r	This study
pRE-*ippR*-L192G	pRESQ harboring *ippR*-L192G; Str^r	This study
pRE-*ippR*-V218G	pRESQ harboring *ippR*-V218G; Str^r	This study
pRE-*ippR*-W257G	pRESQ harboring *ippR*-W257G; Str^r	This study
pRE-*ippR*-W259G	pRESQ harboring *ippR*-W259G; Str^r	This study
pRE-*ippR*-H297G	pRESQ harboring *ippR*-H297G; Str^r	This study
pRE-*ippR*-A300G	pRESQ harboring *ippR*-A300G; Str^r	This study
pRE-*ippR*-K382G	pRESQ harboring *ippR*-K382G; Str^r	This study
pRE-*ippR*-T384G	pRESQ harboring *ippR*-T384G; Str^r	This study
pET-29a (+)	Expression vector; Km^r	Novagen
pET-*ippR*	pET-29a (+) harboring *ippR*; Km^r	This study
pET-V188G	pET-29a (+) harboring *ippR*-V188G; Km^r	This study
pET-L192G	pET-29a (+) harboring *ippR*-L192G; Km^r	This study
pET-V218G	pET-29a (+) harboring *ippR*-V218G; Km^r	This study
pET-W257G	pET-29a (+) harboring *ippR*-W257G; Km^r	This study
pET-W259G	pET-29a (+) harboring *ippR*-W259G; Km^r	This study
pET-H297G	pET-29a (+) harboring *ippR*-H297G; Km^r	This study
pET-A300G	pET-29a (+) harboring *ippR*-A300G; Km^r	This study
pET-K382G	pET-29a (+) harboring *ippR*-K382G; Km^r	This study
pET-T384G	pET-29a (+)harboring *ippR*-T384G; Km^r	This study
pME6522	pVS1-p15A *E. coli*- *Sphingomonas histidinilytica* shuttle vector for transcriptional *lacZ* fusion and promoter probing, Tc^r	(27)
pME6522-P_{IPP1}	128 bp fragment, upstream of TSS, directionally cloned into pME6522, Tc^r	This study

[a] Str^r, Streptomycin resistant; Km^r, kanamycin resistant; Tc^r, tetracycline resistant.

33 **Table S3 Oligonucleotides used in this study**

Primer	Sequence (5′→3′)	Purpose
ippR-upF	CTATGACATGATTACGAATTCTCCTTCGAGACGATCGGG	Construction of plasmid pK18-*ippR*
ippR-upR	GTGCCTGTCGATCCCGGGTCACTCAGTCCAGTGAGGCACC	
ippR-downF	TGACCCGGGATCGACAGG	Construction of plasmid pK18-*ippR*
ippR-downR	CAGGTCGACTCTAGAGGATCCCTCGTGCAGAGCCACCTG	
ippR-hbF	ACCGAGCTCAGATCTACTAGTGCCAGGGACGGCCCGTTC	Construction of plasmid pRE-*ippR*
ippR-hbR	ACACTGGCGGCCGTTACTAGTTCGCGTGCCTGTCGATCCCG	
q16S-F	GTAACACGTGGGTGATCTGCC	qRT-PCR
q16S-R	CGTGTCTCAGTCCCAGTGTG	
qA1-F	CGATGAGACGATCACCGATCTC	qRT-PCR
qA1-R	CGAACACTTCTCGTTGCGC	
qA2-F	CTCCAGCGAGCAACATCAC	qRT-PCR
qA2-R	CGAGCACGACCATGTGGTC	
2558-A1-F	CGTGTAGGTCACCTCCACC	Transcriptional unit analysis
2558-A1-R	CCCTTGCGCAGTGTGACG	
A1-2560-F	ACAAGGAACCACAGGGGC	Transcriptional unit analysis
A1-2560-R	TGCGTCTGCCCTGGCGCT	
2560-A2-F	GTGCGCGGGTCGGCGAGC	Transcriptional unit analysis
2560-A2-R	CGACAAGGAGTCCGAGACCCT	
A2-R-F	GGTCGACGGTGCAAGCCA	Transcriptional unit analysis
A2-R-R	CCGCGACGGAACACATCT	
TSS-A-1	GCTCGCTGGAGAGGATGTTCACGCC	5′-RACE
TSS-A-2	CGAGGGTCTCGGACTCCTTGTCGAGAG	
TSS-A-3	CGACAAGGAGAGGAAGGCGCTGACGG	
ippR-F	TAAGAAGGAGATATACATATGGTGCCTCACTGGACTGAGAAACTGTCC	Construction of plasmid pET-*ippR*
ippR-R	GTGGTGGTGGTGGTGCTCGAGGCGCGCTGCTGTCAGCCA	
V188G-F	CCGACTGGATCTGGGGCACCAGTTGCTCCGT	Construction of plasmid pET-V188G
V188G-R	ACGGAGCAACTGGTGCCCCAGATCCAGTCGG	

L192G-F	GATCTGGTGCACCAGTTGGGGCGTGGGGAGGATGTC	Construction of plasmid pET-L192G
L192G-R	GACATCCTCCCCACGCCCCAACTGGTGCACCAGATC	
V218G-F	CCGCACATCGCCGTTGGGGCCTGGACCGGCCCA	Construction of plasmid pET-V218G
V218G-R	TGGGCCGGTCCAGGCCCCAACGGCGATGTGCGG	
W257G-F	GATTGCTCCCCATGCCCCCACCGCCTGAAGGCC	Construction of plasmid pET-W257G
W257G-R	GGCCTTCAGGCGGTGGGGGCATGGGGAGCAATC	
W259G-F	CAGGCGGTGTGGGCAGGGGGAGCAATCGGTCCC	Construction of plasmid pET-W259G
W259G-R	GGGACCGATTGCTCCCCCTGCCCACACCGCCTG	
H297G-F	GGTTTCCGGAGAAGTGGGACCGAAGCCAAGGCG	Construction of plasmid pET-H297G
H297G-R	CGCCTTGGCTTCGGTCCCACTTCTCCGGAAACC	
A300G-F	GAGAAGTCATACCGAAGGGAAGGCGGTGGAACGG	Construction of plasmid pET-A300G
A300G-R	CCGTTCCACCGCCTTCCCTTCGGTATGACTTCTC	
K382G-F	GATAGGTCACCGTGTTTGCCGAGATGTGTTCCGTCG	Construction of plasmid pET-K382G
K382G-R	CGACGGAACACATCTCGGCAAACACGGTGACCTATC	
T384G-F	GTTGATGCGATAGGTCACGGGGTTCTTCGAGATGTGTTC	Construction of plasmid pETT384G
T384G-R	CAACTACGCTATCCAGTGCCCCAAGAAGCTCTACACAAG	
F_{IPP1}-F	CGGAAGTGTCCTTCGATCGA	EMSA
F_{IPP1}-R	CTGACCCGGGATCGACAG	
ippA1-F	CGCCGCCGATATCGTCCG	EMSA
ippA1-R	CGAACACTTCTCGTTGCGC	
F_{IPP1}-1-F	CGGAAGTGTCCTTCGATCGA	EMSA
F_{IPP1}-1-R	TAGAACACAGCCGGTCCTCC	
F_{IPP1}-2-F	TTATCGGAGGACCGGCTGTG	EMSA
F_{IPP1}-2-R	GGAACCAACAGGGACGGC	
F_{IPP1}-3-F	TTCTAGGACACAATTAGAACGCACC	EMSA
F_{IPP1}-3-R	CGGGCCGATGGGTTGGTC	
F_{IPP1}-4-F	GCGCAGCCTAAGGTTGGC	EMSA
F_{IPP1}-4-R	TCCGTATCCACGAGTGGCT	
F_{IPP1}-5-F	ACCGGATGCGACCTGCGC	EMSA

F_{IPP1}-5-R	CTGACCCGGGATCGACAG	
F_{IPP1}-6-F	ACCAATGCGCAGGTCG	EMSA
F_{IPP1}-6-R	TGAGGATGGAGCGCCGGG	
M1-1-F	ACCGGATGCGATCTGCGCATTGGTGCGCAG	EMSA
M1-1-R	ACAGCAGCGCGCTGACCCGGGATCGACAG	
M1-2-F	ACCGGATGCGACTTGCGCATTGGTGCGCAG	EMSA
M1-2-R	ACAGCAGCGCGCTGACCCGGGATCGACAG	
M1-F	ACCGGATGCGATTTGCGCATTGGTGCGCAG	EMSA
M1-R	ACAGCAGCGCGCTGACCCGGGATCGACAG	
M2-1-F	ACCGGATGCGACCTGTGCATTGGTGCGCAG	EMSA
M2-1-R	ACAGCAGCGCGCTGACCCGGGATCGACAG	
M2-2-F	ACCGGATGCGACCTGCACATTGGTGCGCAG	EMSA
M2-2-R	ACAGCAGCGCGCTGACCCGGGATCGACAG	
M2-F	ACCGGATGCGACCTGTACATTGGTGCGCAG	EMSA
M2-R	ACAGCAGCGCGCTGACCCGGGATCGACAG	
MT-F	ACCGGATGCGATTTGTACATTGGTGCGCAG	EMSA
MT-R	ACAGCAGCGCGCTGACCCGGGATCGACAG	
ippA2-6522-F	CGGAATTCCCCCGGGATCGACAGGCAC	Construction of plasmid pME6522-P_{IPP1}
ippA2-6522-R	AACTGCAGCTAGGACACAATTAGAACGCACCG	

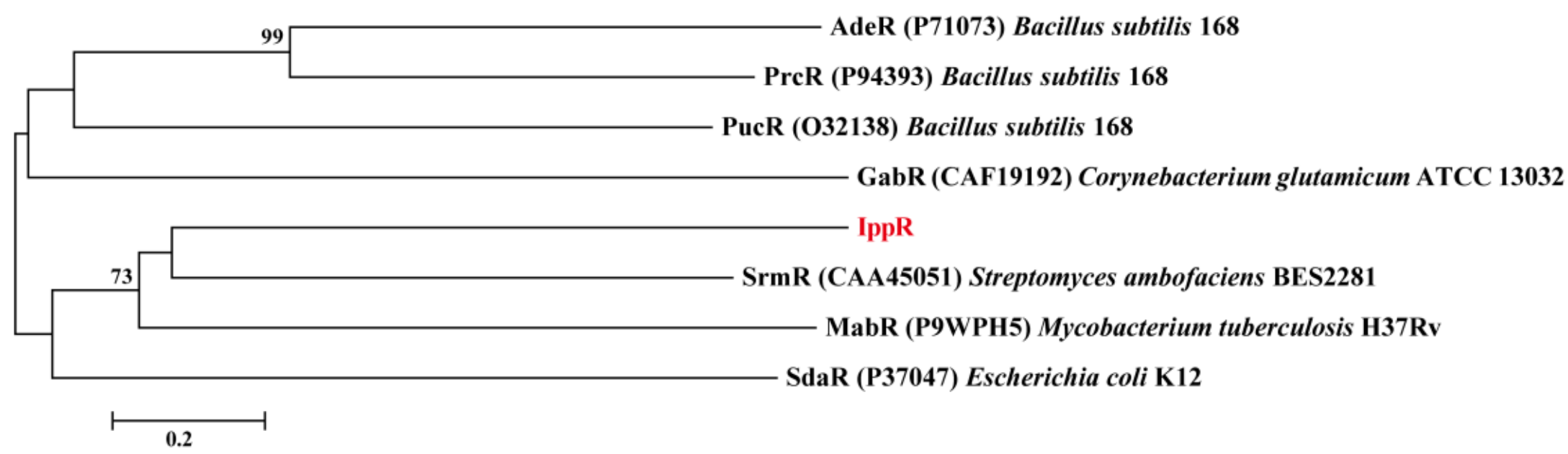

Fig. S1. Phylogenetic analysis of PcaR and PucR family transcriptional regulators by neighbor-joining method. Bootstrap values (%) are indicated at the branch nodes, the scale bar represents 0.2 substitutions per site. The accession numbers are in parentheses.

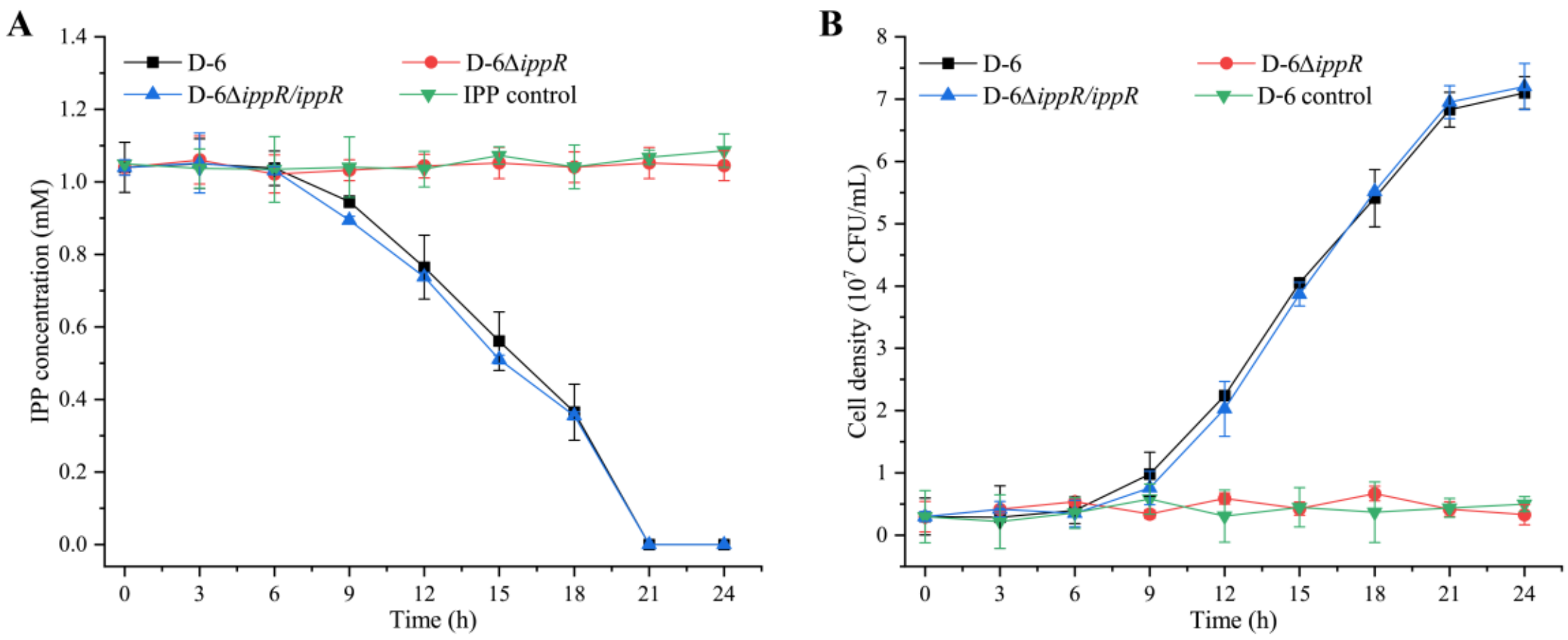

Fig. S2. Degradation (A) and growth (B) of 1.04 mM IPP by wild-type strain D-6, the *ippR* knockout strain D-6*ΔippR* and *ippR*-complementary strain D-6*ΔippR/ippR*. The tested strains D-6, D-6*ΔippR* and D-6*ΔippR/ippR* were cultured in LB medium, harvested by centrifugation, and washed three times with MSM. The washed cells were suspended in MSM containing 1.04 mM IPP to an initial density of approximately 3.0 × 10^6 CFU/mL. The concentration of residual IPP was determined every 3 h by HPLC after filtration through 0.22-μm filters. Simultaneously, cell growth was determined by the colony counting method. Error bars represent the standard deviation (SD) of three replicates.

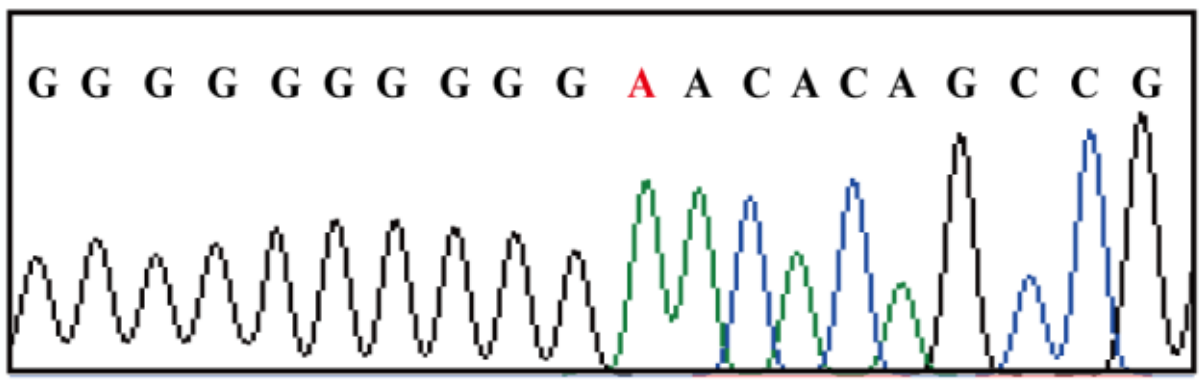

Fig. S3. The TSS determination of the *IPP1* operon by 5′-RACE. The red letter A indicates its TSS.

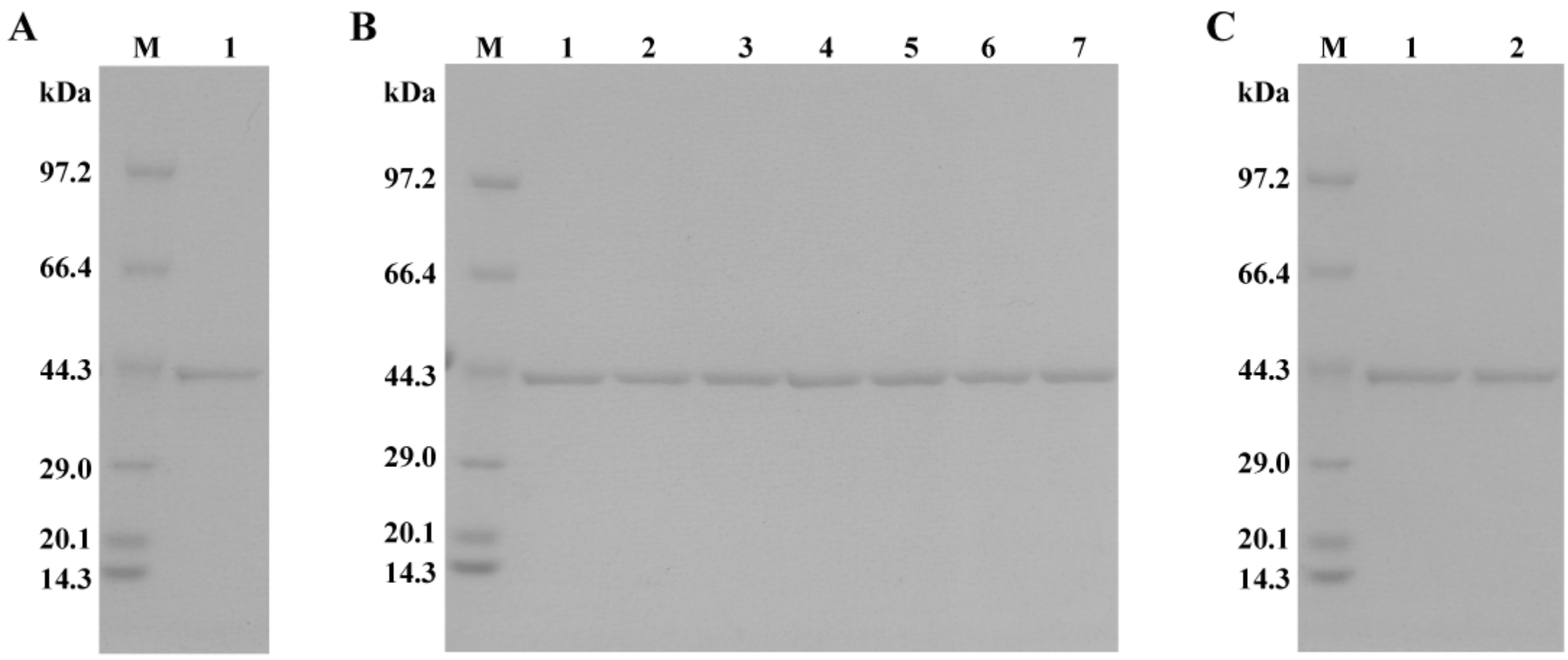

Fig. S4. SDS-PAGE analysis of the purified proteins. (A) SDS-PAGE analysis of the purified IppR. Lane M, protein marker; lane 1, IppR. (B) Lane M, protein marker; lane 1, IppR-V188G; lane 2, IppR-L192G; lane 3, IppR-V218G; lane 4, IppR-W257G; lane 5, IppR-W259G; lane 6, IppR-H297G; lane 7, IppR-A300G. (C) Lane M, protein marker; lane 1, IppR-K382G; lane 2, IppR-T384G.

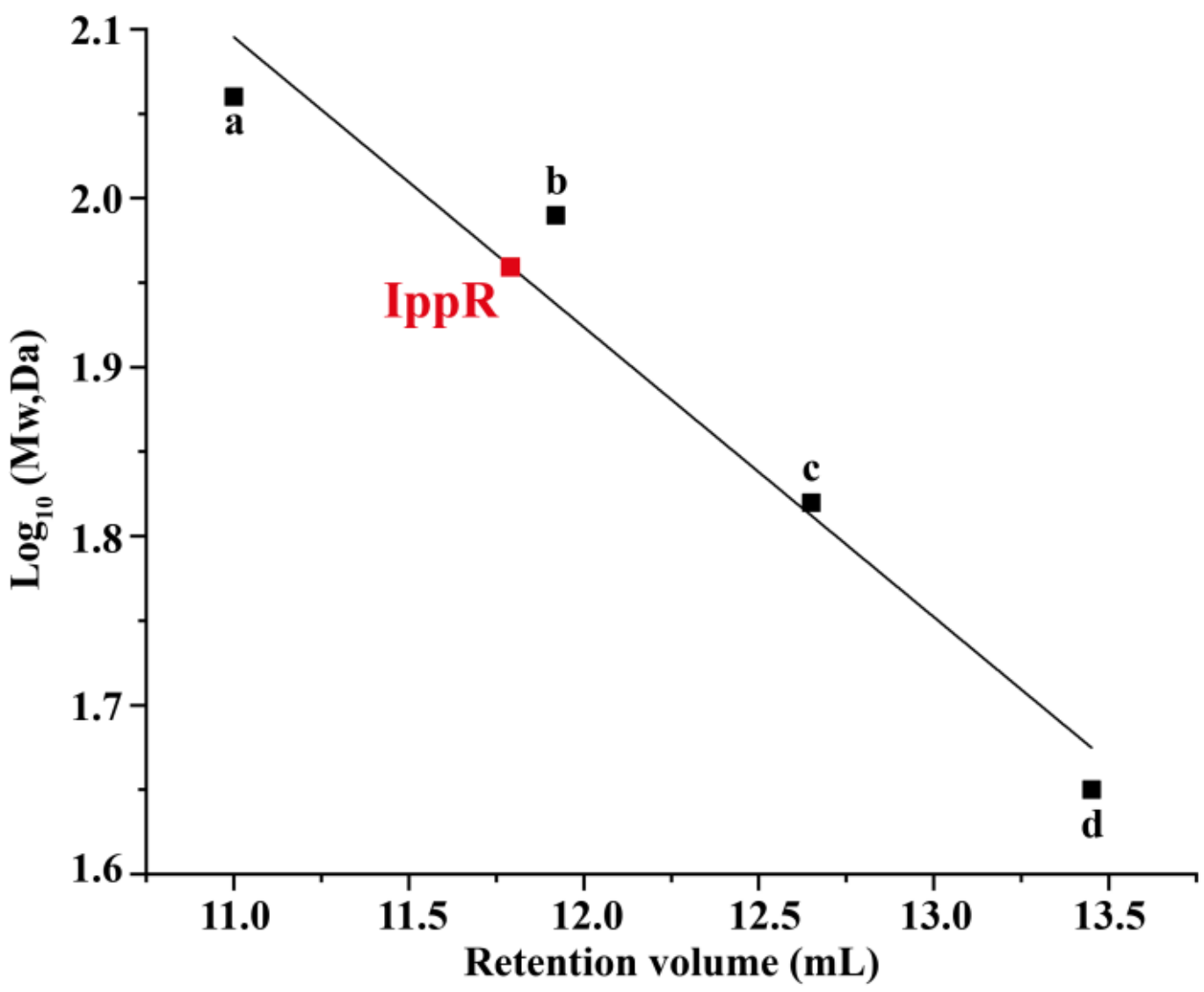

Fig. S5. Determination of native IppR based on the standard curve using gel filtration. The standard proteins include *β*-galactosidase (a, 116 kDa, 11.00 mL), phosphorylase b (b, 97 kDa, 11.92 mL), bovine serum albumin (c, 66 kDa, 12.65 mL), and ovalbumin (d, 45 kDa, 13.45 mL) were used. The standard curve was fitted as $Log_{10}(Mw) = -0.1716 V + 3.9828$ (R^2=0.9536). The elution volume of native IppR was determine to be 11.79 mL. The molecular weight calculated from the generated standard curve was 91.1 kDa, which was close to the theoretical molecular weight of IppR dimer (90.0 kDa).

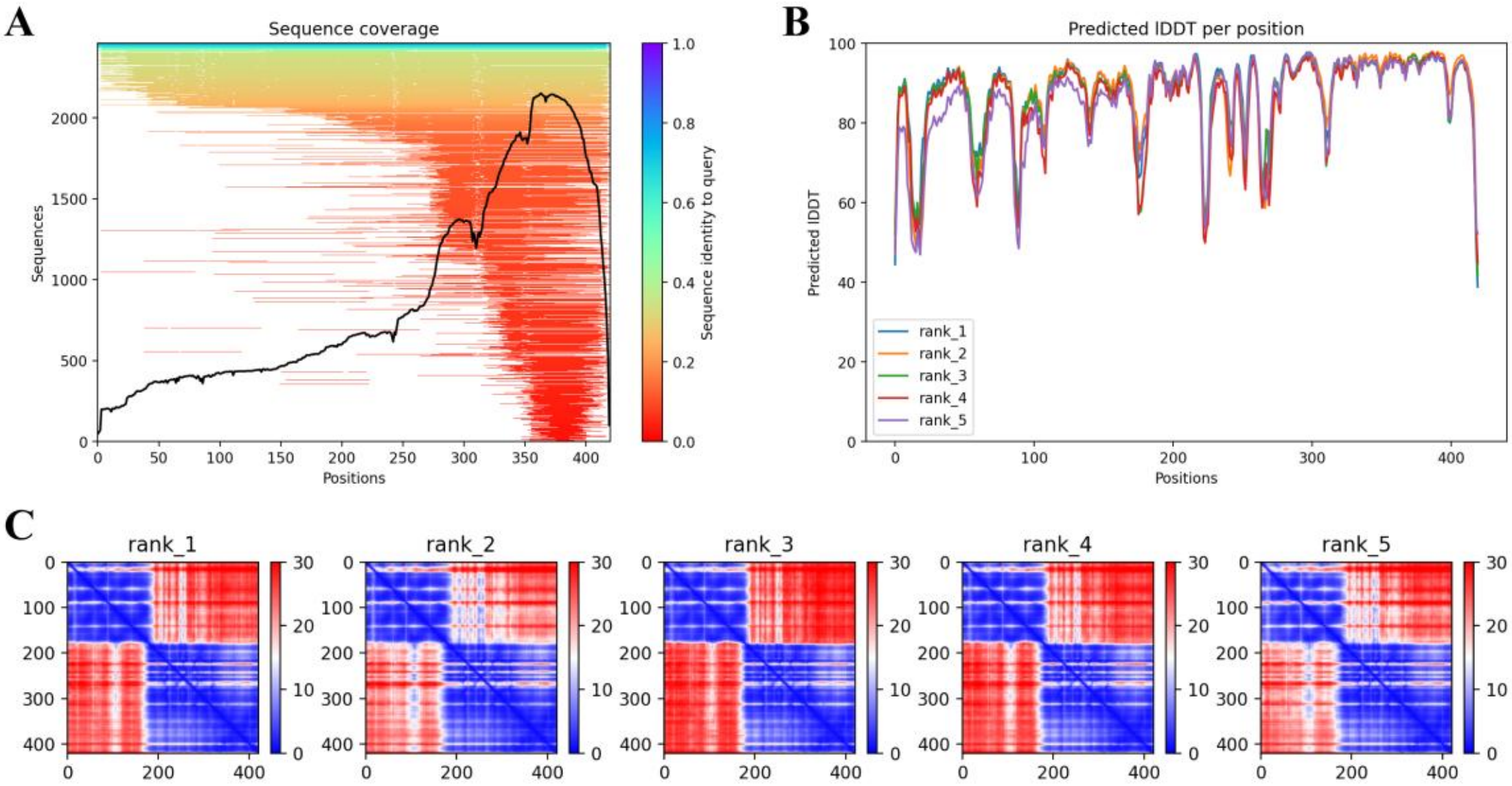

Fig. S6. Evaluation of the predicted structural models of IppR. (A) Sequence coverage of the multiple sequence alignment for IppR. (B) Per-residue pLDDT scores of the five predicted models. Regions with pLDDT < 50 were considered to have low confidence. (C) Predicted aligned error (PAE) plots of the five models. The mean pLDDT scores of models I, II, III, IV, and V were 87.1, 85.5, 87.6, 88.0, and 85.2, respectively.

Supplemental Text

IPC, 2-isopropylhydroquinone and its downstream metabolites show no effector activity of IppR

qRT-PCR and *β*-galactosidase activity assays were conducted to verify that IPC, 2-isopropylhydroquinone and its downstream metabolites were not the effector of IppR.

1. qRT-PCR: the transcriptional levels of *ippA1* and *ippA2* were evaluated in cells of strains D-6, D-6*ΔipcH* (the *ipcH* knockout strain which lost its ability to degrade IPC) grown on glucose, IPC or 2-isopropylhydroquinone using qRT-PCR. In strain D-6, the transcription levels of *ippA1* and *ippA2* were 30-fold and 33-fold higher under IPC treatment than those cultivated with glucose or 2-isopropylhydroquinone (Fig. ST1A). These data indicated that IPC and its hydrolysis product IPP might act as the effectors of IppR, while 2-isopropylhydroquinone and its downstream metabolites did not. Additionally, the expression of *ippA1* and *ippA2* remained at low levels in D-6Δ*ipcH* under both glucose and IPC treatments, demonstrating that IPC is not a direct effector of IppR.

2. *β*-Galactosidase activity assay: the reporter strains D-6-P_{IPP1} and D-6*ΔipcH*-P_{IPP1} were treated with glucose, IPC or 2-isopropylhydroquinone. Strain D-6-P_{IPP1} exhibited low *β*-galactosidase activity in the presence of glucose (33 Miller units) and 2-isopropylhydroquinone (43 Miller units), whereas IPC significantly increased its activity to 374 Miller units (Fig. ST1B). Consistent with the results of qRT-PCR analysis, this finding suggested potential effector roles of IPC and IPP, excluding 2-isopropylhydroquinone and its degradation metabolites. Meanwhile, strain D-6*ΔipcH*-

P_{IPP1} maintained low enzymatic activity regardless of glucose or IPC supplementation, further confirming that IPC cannot serve as an IppR effector (Fig. ST1B).

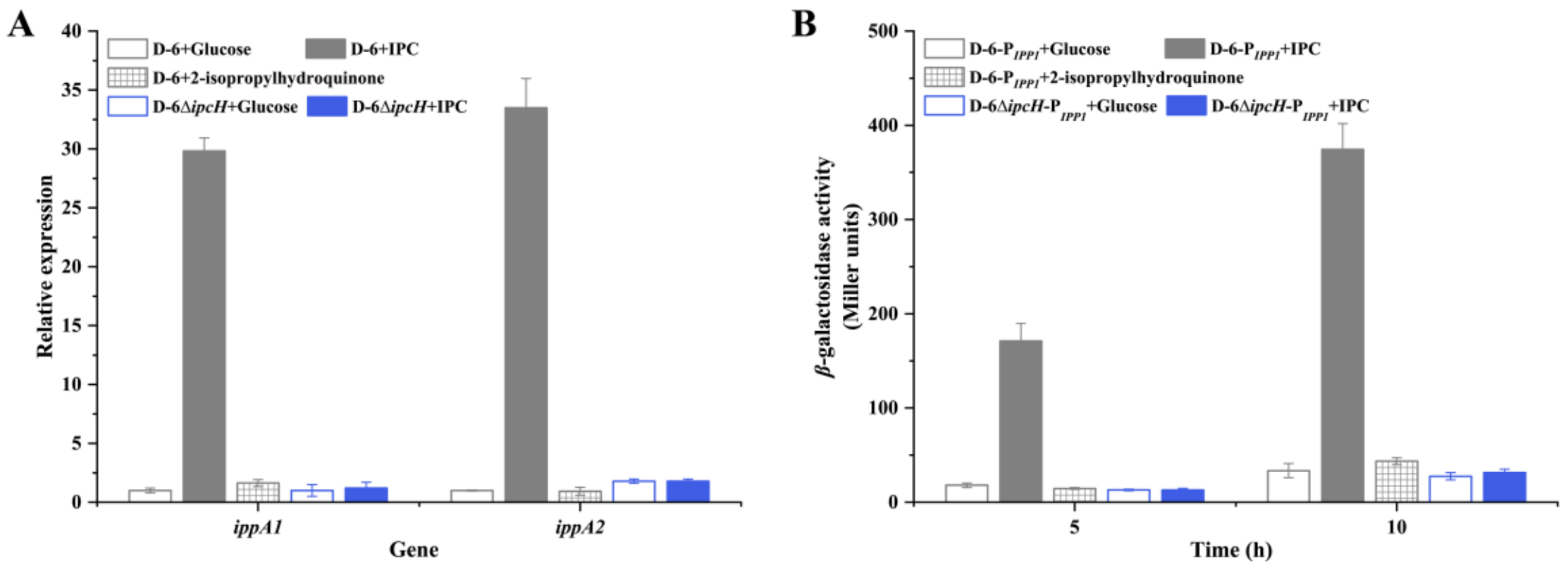

Fig. ST1. The regulatory effect of IPC, 2-isopropylhydroquinone and its downstream metabolites on the activity of IppR. (A) Transcriptional analysis of *ippA1* and *ippA2* in strains D-6 and *ipcH* knockout strain D-6*ΔipcH* in the presence of 1.04 mM glucose, IPC or 2-isopropylhydroquinone. The transcriptional level of the 16S rRNA gene was used as an internal standard, and the data in each column were calculated by the $2^{-\Delta\Delta CT}$ threshold cycle (C_T) method using three replicates. (B) The *β*-galactosidase activity assay was performed with strain D-6-P_{IPP1} and D-6*ΔipcH*-P_{IPP1} in the presence of 1.04 mM glucose, IPC or 2-isopropylhydroquinone.
